# Therapy with Plasma Purified Alpha1-Antitrypsin (Prolastin^®^) Induces Time-Dependent Changes in Plasma Levels of MMP-9 and MPO

**DOI:** 10.1371/journal.pone.0117497

**Published:** 2015-01-30

**Authors:** Janine Koepke, Marc Dresel, Severin Schmid, Timm Greulich, Björn Beutel, Bernd Schmeck, Claus Franz Vogelmeier, Sabina Janciauskiene, Andreas Rembert Koczulla

**Affiliations:** 1 Department of Medicine, Pulmonary and Critical Care Medicine, University Medical Center Giessen and Marburg, Philipps-University, Member of the German Center for Lung Research (DZL), Baldingerstraße 1, Marburg, Germany; 2 Institute for Lung Research, Universities of Giessen and Marburg Lung Center, Philipps-University Marburg, Member of the German Center for Lung Research (DZL), Hans-Meerwein-Straße 2, Marburg, Germany; 3 Department of Respiratory Medicine, Hannover Medical School, Member of the German Center for Lung Research (DZL), Feodor-Lynen-Straße 23, Hannover, Germany; RWTH Aachen, GERMANY

## Abstract

The common Z mutation (Glu342Lys) of α1-antitrypsin (A1AT) results in the polymerization and intracellular retention of A1AT protein. The concomitant deficiency of functional A1AT predisposes PiZZ subjects to early onset emphysema. Clinical studies have implied that, among the biomarkers associated with emphysema, matrix metalloproteinase 9 (MMP-9) is of particular importance. Increased plasma MMP-9 levels are proposed to predict the decline of lung function as well as greater COPD exacerbations in A1AT deficiency-associated emphysema. The aim of the present study was to investigate the effect of A1AT therapy (Prolastin) on plasma MMP-9 and myeloperoxidase (MPO) levels. In total 34 PiZZ emphysema patients were recruited: 12 patients without and 22 with weekly intravenous (60 mg/kg body weight) A1AT therapy. The quantitative analysis of A1AT, MMP-9 and MPO was performed in serum and in supernatants of blood neutrophils isolated from patients before and after therapy. Patients with Prolastin therapy showed significantly lower serum MMP-9 and MPO levels than those without therapy. However, parallel analysis revealed that a rapid infusion of Prolastin is accompanied by a transient elevation of plasma MMP-9 and MPO levels. Experiments with freshly isolated blood neutrophils confirmed that therapy with Prolastin causes transient MMP-9 and MPO release. Prolastin induced the rapid release of MMP-9 and MPO when added directly to neutrophil cultures and this reaction was associated with the presence of IgA in A1AT preparation. Our data support the conclusion that changes in plasma levels of MMP-9 and MPO mirror the effect of Prolastin on blood neutrophils.

## Introduction

Alpha1-Antitrypsin (A1AT), also referred to as alpha1-proteinase inhibitor or SERPINA1, is an acute phase protein and the prototypical member of the SERPIN (serine proteinase inhibitor) family of protease inhibitors. Plasma concentrations of A1AT range from 0.9 to 2 mg/ml when measured by nephelometry. The circulating levels of A1AT increase rapidly in response to inflammation or infection [1]. Tissue concentrations of A1AT may increase as much as 11-fold as a result of local synthesis by resident or invading inflammatory cells but it is cleared with a half-life of three to five days [2,3].

The level of A1AT is controlled by a pair of co-dominant alleles. The prevalence of the major A1AT variants (PI*M, PI*Z, and PI*S) is reported in many surveys as gene frequencies. The highest prevalence of the PI*Z variant is recorded in northern and western European countries (mean gene frequency 0.0140), peaking in southern Scandinavia, the Netherlands, the UK, and northern France (gene frequency >0.0200) [4].

The Z A1AT results from the mutation (Glu342Lys) in exon V of the gene. This mutation causes a change in A1AT protein folding leading to polymerization and intracellular retention. Because hepatocytes are the main source for A1AT, polymers of A1AT can promote liver damage with a variable clinical presentation, from neonatal hepatitis to liver cirrhosis and hepatocellular carcinoma in adults. On the other hand, the lack of circulating protein predisposes to the development of early-onset emphysema [5,6]. Extra-hepatic A1AT polymerization also occurs [7,8]. Current data demonstrate that cigarette smoke promotes polymerization of Z A1AT via oxidation of the protein [9].

Augmentation therapy with pooled human plasma A1AT was introduced 30 years ago to treat emphysema patients with ZZ deficiency of A1AT. This therapy is typically started in patients older than 18 years who have moderate emphysema (FEV1 <65% of expected) and is continued indefinitely. Most of the patients receiving weekly intravenous infusions of 3–5 g clinical grade A1AT (60 mg/kg body weight) [10].

Proof of efficacy of augmentation therapy has been attempted in several clinical trials that explored as the primary outcomes lung function tests and computer tomography densitometry [11]. The overall results from the existing clinical trials support the efficacy of A1AT therapy. The experience with A1AT therapy suggests that it is safe, with few and usually well tolerated side effects, and that this therapy reduces the decline in lung density [12,13]. Stockley and colleagues have shown that augmentation therapy (60 mg/kg weekly for four weeks) favourably changes sputum inflammatory milieu i.e. reduces sputum elastase activity and levels of the leukotriene B4, IL-8 and myeloperoxidase (MPO) [14]. Furthermore, Prolastin (2.5 mg per nostril) has been found to inhibit nasal IL-8 release in response to LPS challenge and to inhibit LPS-induced TNFα and IL-1β release from monocytes and IL-8 release from neutrophils, *in vitro* [15]. Previously we have measured plasma levels of A1AT polymers, cytokines (IL-1β, IL-6, and TNFα), chemokines (IL-8 and MCP-1) and VEGF in patients undergoing weekly augmentation therapy- just before intravenous A1AT (Prolastin) infusion, as well as one and three days after therapy. Our results revealed significant fluctuations in plasma cytokine and A1AT polymer concentrations, pointing out that potentially unacknowledged immunologic properties are carried out by weekly infusion of A1AT [16].

Matrix metalloproteinase-9 (MMP-9) is one of the proteinases that have received considerable attention in COPD [17–19]. For example, Higashimoto and colleagues examined multiple biomarkers among 96 COPD patients, finding that only MMP-9 and C-reactive protein were statistically significantly associated with declines in FEV1 [18]. Omachi and co-authors suggested that MMP-9 may be a valuable biomarker for the progression of emphysema with A1AT deficiency [20]. Interestingly, a study by Stone *et al*. has found that A1AT deficient patients have higher sputum concentrations of IL-8 and LTB4 compared with usual COPD, but lower levels of MPO and absolute neutrophil counts [21]. Therefore, we wanted to continue investigations on the effects of weekly Prolastin therapy, with specific focus on changes in serum levels of MMP-9 and MPO.

## Materials and Methods

### Study participants and Ethics Statement

This study enrolled 35 patients, clinically diagnosed for PiZZ A1AT-related emphysema. Every patient answered a questionnaire regarding the symptoms, health status, medical and smoking history. Individuals who had recent acute exacerbations (within the last three months) or were taking oral steroids were excluded from the study. The informed consent document has been signed by all patients. The study was approved by the Philipps University of Marburg local ethics committee (59/06 and 148/12). Out of 34 patients, 22 were under weekly intravenous (60 mg/kg body weight) therapy with human purified A1AT (Prolastin, Grifols) and 12 patients were without A1AT therapy. The mean forced expiratory volume in one second was 43.5% and 60.1% in treated and non-treated patients with A1AT deficiency, respectively (Table 1).

**Table 1:** Patient characteristics/ serum measurements.

PiZZ patients	Prolastin therapy(day 1 pre/post, n = 22)	Prolastin therapy(day 3, n = 12)	No Prolastin therapy(n = 12)	One patientwith Prolastin therapy(weekly timecourse)	One patientno Prolastin therapy(weekly timecourse)
Age in years, mean (SD)	55 (8.5)	58 (9.9)	51.9 (8.2)	51	60
Sex, F/ M	5/ 17	3/ 9	4/ 8	0/ 1	0/ 1
FEV_1_ in %, mean (SD)	43.5 (15.2)	44.4 (15.7)	60.1 (20.2)	30.2	56,9
Smoking ex/ current	19/ 0	10/0	8/ 0	1/ 0	0/ 0
Pack-years, mean (SD)	13.2 (6.7)	12.8 (6.8)	15.9 (15.9)	10	0
GOLD stageII/ III/ IV	9/ 7/ 6	6/ 3 / 3	9/ 1/ 2	0/ 1/ 0	1/ 0/ 0

### Study design

From PiZZ patients receiving weekly intravenous Prolastin therapy blood samples were obtained just before therapy (pre, n = 22), two hours (post, n = 22) and three days after therapy (day 3, n = 12). From PiZZ patients, who were not on the Prolastin therapy, blood samples were obtained only once (Table 1). From one PiZZ patient (GOLD III) receiving Prolastin therapy and from one non-treated PiZZ patient (GOLD II), blood sample and blood neutrophils were obtained every day during one week. From the patient receiving Prolastin additional samples were taken before (pre) and two hours after (post) A1AT infusion (Table 1). For *ex vivo* experiments, blood neutrophils were isolated just before (pre, n = 8) and two hours (post, n = 8) after therapy with Prolastin as well as from non-treated A1AT patients (n = 8, Table 2).

**Table 2:** Patient characteristics/ degranulation PMNs.

PiZZ patients	Prolastin therapy(pre/post, n = 8)	No Prolastin therapy(n = 8)
Age in years, mean (SD)	65.4 (8.8)	49 (12.9)
Sex, F/ M	3/ 5	3/ 5
FEV_1_ in %, mean (SD)	51 (14.9)	92.1 (35.3)
Smoking ex/ current	0/ 6	0/ 4
Pack years, mean (SD)	8.9 (6.6)	5.8 (7.3)
GOLD stage0/ I/ II/ III/ IV	0/ 0/ 4/ 4/ 0	5/ 0/ 2/ 0/ 1

### A1AT quantification by nephelometry

Serum samples were analyzed for the levels of A1AT by nephelometry (Behring Nephelometer 2 BN2, Siemens) in the routine laboratory of the Marburg University Hospital.

### Analysis of serum MMP-9 and MPO by ELISA

For the quantitative determination of serum MMP-9 and MPO we used commercial available ELISA kits (Human DuoSet R&D Systems, Abingdon, UK). Serum samples were diluted 1:500 or 1:1000 with dilution buffer and measured in duplicates using Tecan infinite F200pro. For MPO the standard range was between 62.5–4000 pg/ml with a sensitivity of 62.5 pg/ml. For MMP-9 the standard range was between 31.25–2000 pg/ml with a sensitivity of 31.25 pg/ml.

### Analysis of MMP-9 and MPO in neutrophil culture supernatants by ELISA

Neutrophils were isolated from the peripheral blood of PiZZ A1AT patients before (pre, n = 8) and two hours after (post, n = 8) therapy with Prolastin using Polymorphprep (Axis-Shield PoC AS, Oslo, Norway) as previously described [22]. The neutrophil purity and cell viability exceeded 95%. Purified neutrophils (1×10^6^ cells/ml) were resuspended in RPMI medium supplemented with 10% volume/volume fetal calf serum and incubated with or without 1 mg/ml A1AT (Prolastin) for 4 hours at 37°C, 5% CO_2_. The cell culture supernatants were collected and analyzed for the degranulation of MMP-9 and MPO by ELISA kits (Human DuoSet R&D Systems, Abingdon, UK).

### Western blot analysis

Neutrophils were isolated from the peripheral blood of two PiZZ A1AT patients every day in a week course (Table 1, one patient with and one patient without Prolastin therapy) as described in the section above. Isolated cells were washed with PBS, lysed and the protein concentration was determined (BCA assay, Pierce BCA Protein Assay Kit, Thermo Scientific). Equal amounts of analyzed protein were separated on 7.5% sodium dodecyl sulfate polyacrylamide gel electrophoresis (SDS-PAGE). Proteins were transferred to a polyvinylidene fluoride (PVDF) membrane and detected using the primary anti-A1AT antibody (polyclonal IgG antibody, Bethyl Laboratories, Inc., USA; dilution 1:10000 in Tris-Buffered Saline and Tween with 5% milk powder). Membranes were incubated over night at 4°C, washed and incubated for 1 hour at room temperature with secondary anti-goat antibody (HRP conjugated, Sigma Aldrich, St Louis, USA; diluted at 1:20000 in Tris-Buffered Saline and Tween with 3% milk powder). Protein loading was monitored by stripping and re-probing the membranes with β-actin (primary monoclonal anti-actin antibody, IgG, produced in rabbit, Merck Millipore, Germany, diluted 1:1000 in Tris-Buffered Saline and Tween with 3% milk powder, incubation over night at 4°C; secondary anti-rabbit antibody, IgG, produced in chicken, HRP conjugated, dilution 1:20000 in Tris-Buffered Saline and Tween with 3% milk powder for one hour at room temperature, Abcam, UK). ECL Western blotting method was used for detection of immobilized specific antigens (GE healthcare, Buckinghamshire, UK) applying Science Imaging, ChemoCam system. Each individual Western blot was directly developed for 10 min.

### A1AT Preparations

Purified human plasma pooled A1AT (Prolastin, Grifols) was used for *in vitro* experiments. To remove high molecular mass proteins, specifically IgA [16,23,24] Prolastin was first diluted in phosphate-buffered saline (PBS) and repurified by ultrafiltration using a centricon-100 kDa and centricon-30 kDa cutoff. The IgA-free preparation of A1AT was subjected to quantitative analysis using bicinchoninic acid assay (BCA assay, Pierce BCA Protein Assay Kit, Thermo Scientific) and to qualitative evaluation using Western blot analysis with specific anti-A1AT and anti-IgA antibody.

### Statistical analysis

Statistical analysis was performed using GraphPad Prism 5.03 software (GraphPad Software, Inc, La Jolla, CA). Data is presented as mean value and standard deviation. In order to test for normality the D’Agostino—Pearson-omnibus normality test was performed. Normal distributed values were compared using student’s paired t-test or unpaired t-test, respectively. In the event of a failing normality-test paired values were matched using the Wilcoxon-signed-rank test or, if unpaired, using the Mann-Whitney test. Correlations were calculated using the Pearson’s and Spearman’s correlation test.

## Results

### Serum concentrations of A1AT in PiZZ patients with and without Prolastin therapy

**Fig 1:**
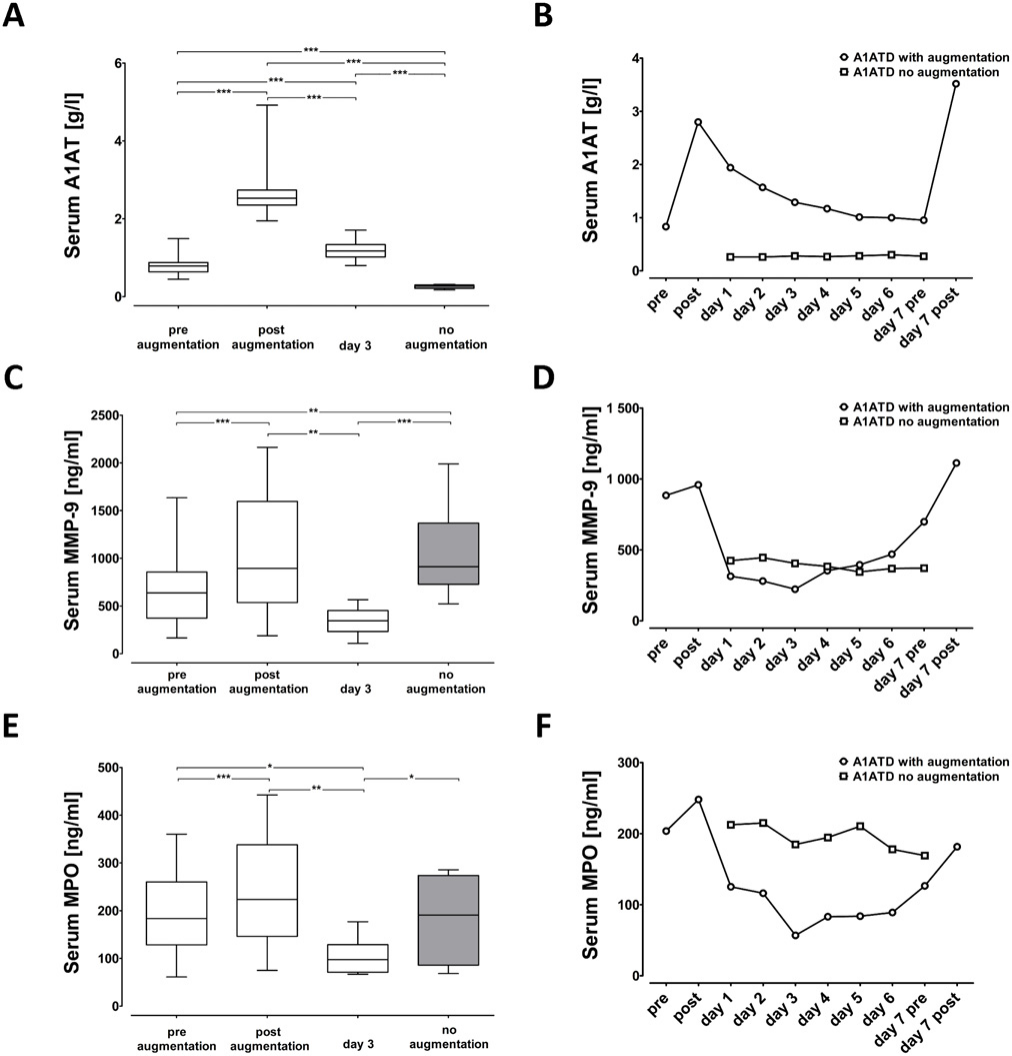
Quantitative analysis of serum A1AT, MMP-9 and MPO. Measurement of (***A***) A1AT, (***C***) matrix metalloproteinase 9 (MMP-9) and (***E***) myeloperoxidase (MPO) levels in serum from augmented and non-augmented PiZZ A1ATpatients. From augmented patients serum samples were obtained at three different time points: before (pre, n = 22), two hours after (post, n = 22) and on day three after augmentation therapy (day 3, n = 12). From the non-augmented PiZZ A1AT deficient-patients serum samples were obtained only once (no augmentation, n = 12). To follow up the level of (***B***) A1AT as well as the biomarkers (***D***) MMP-9 and (***F***) MPO in a week course analysis, we obtained serum samples every day from one augmented PiZZ A1AT patient (GOLD stage III) and one non-augmented PiZZ A1AT-patient (GOLD stage II). Statistical significance between indicated groups at *** p ≤ 0.001, ** p ≤ 0.01 and * p ≤ 0.05.

As illustrated in Fig. 1A, mean serum levels of A1AT were lower in PiZZ patients without Prolastin therapy than in those with continues therapy [mean (SD): 0.259 (0.04) versus 0.79 (0.22) g/L, respectively; p = 0.0001]. As expected, two hours after Prolastin therapy serum levels of A1AT increased by 3.4-fold [mean (SD): from 0.79 (0.22) to 2.67 (0.63) g/L, p *=* 0.0001]. Serum testing for A1AT on day-3 revealed that A1AT concentration decreased by 2.23-fold relative to day one [mean (SD): 2.67 (0.63) versus 1.19 (0.23) g/L, p = 0.0005]. Nevertheless, levels of A1AT were still significantly higher than those before weekly infusion of Prolastin (Fig. 1). In a single patient serum levels of A1AT were examined every day during one week. As a representative from the group of patients receiving Prolastin therapy, we chose a 52 year old male, ex-smoker (pack years 10) with GOLD stage III COPD (FEV_1_% 31). From the group without Prolastin therapy, serum samples were obtained from a 60 year old male, never-smoker; with GOLD stage II COPD (FEV_1_% 56.9). The week course analysis of serum concentration of A1AT after Prolastin infusion revealed that the level of A1AT initially rose very fast, but dropped after 24 h by about 30.7%, and was followed by a slow decline throughout the week course (Fig. 1B). In the patient without Prolastin therapy, serum concentration of A1AT remained relatively stable over one week (Fig. 1B).

### Serum concentrations of MMP-9 and MPO in PiZZ patients with and without Prolastin therapy

As shown in Fig. 1C, serum levels of MMP-9 increased significantly two hours after A1AT therapy relative to those measured prior to the therapy [mean (SD): post infusion 629.6 (309.08) versus pre infusion 429.4 (154.96) ng/ml, p ≤ 0.0001]. In contrast, three days post therapy serum levels of MMP-9 decreased below the initial level and were significantly lower than those measured directly after A1AT infusion [mean (SD): 334.3 (138) ng/ml versus 629.6 (309.08), respectively, p<0.005]. At all studied time points mean serum levels of MMP-9 were significantly higher in non-augmented than in augmented patients (Fig. 1C). Similarly, therapy with A1AT resulted in a direct induction of serum MPO levels [mean (SD): post infusion 244.29 (101) versus pre infusion 187.95 (81.7) ng/ml, p ≤ 0.0001; Fig. 1D]. Again, as for MMP-9, three days post therapy, levels of MPO decreased and were significantly lower than in non-treated patients (Fig. 1E). Additional investigations using serum from a single patient confirmed that A1AT therapy leads to an immediate elevation, gradual decrease (until day three) and the restoration of serum MPO levels to the starting level at day seven. Notably, no changes in MMP-9 and MPO levels during one week were observed in a single patient without A1AT therapy (Fig. 1D and E).

### Correlations between measured variables

**Fig 2:**
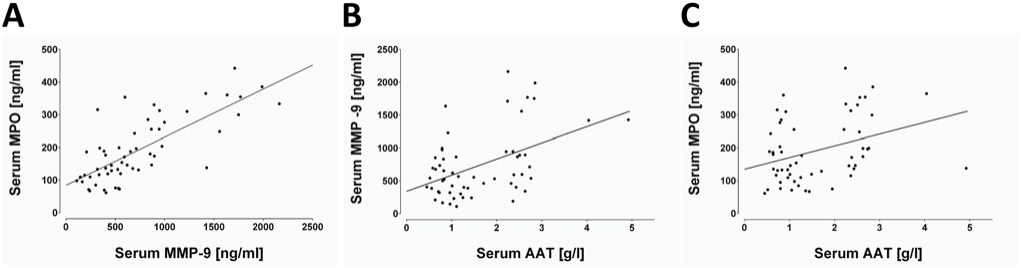
The correlation between serum levels of A1AT, MMP-9 and MPO in PiZZ patients receiving Prolastin therapy. (*A*) Spearman correlation analysis shows a significant positive correlation between the serum levels of MMP-9 and MPO (r = 0.7284, p<0.0001, n = 56); A1AT and MMP-9 (*B*) as well as A1AT and MPO (*C*) (r = 0.34, p = 0.009, n = 56; and r = 0.32, p = 0.016, n = 56, respectively). Line represents linear regression of data (MMP-9 with MPO: slope 0.1471±0.0175, r2 = 0.567; A1AT with MMP-9: slope 245.9±62.5, r2 = 0.223; A1AT with MPO: slope 35.54±12.98, r2 = 0.122).

We next sought to evaluate the relationship between serum levels of A1AT, MMP-9 and MPO in PiZZ patients without and with Prolastin therapy. First of all, in patients not receiving Prolastin no correlations were found between A1AT and MMP-9 or MPO (data not shown). As illustrated in Fig. 2 A, a positive correlation between MMP-9 and MPO was found in all patients receiving Prolastin, independent of whether the sample was obtained before or after (post and day three) therapy (n = 56, r = 0.72, p<0.0001). Furthermore, in treated patients were found weak but significant correlations between serum levels of A1AT and MMP-9, as well as A1AT and MPO (n = 56, r = 0.34, p = 0.009 and p = 0.016, Fig. 2B and C, respectively).

### Changes of MMP-9 and MPO concentrations in supernatants of neutrophil isolated from single patients with and without Prolastin therapy

**Fig 3:**
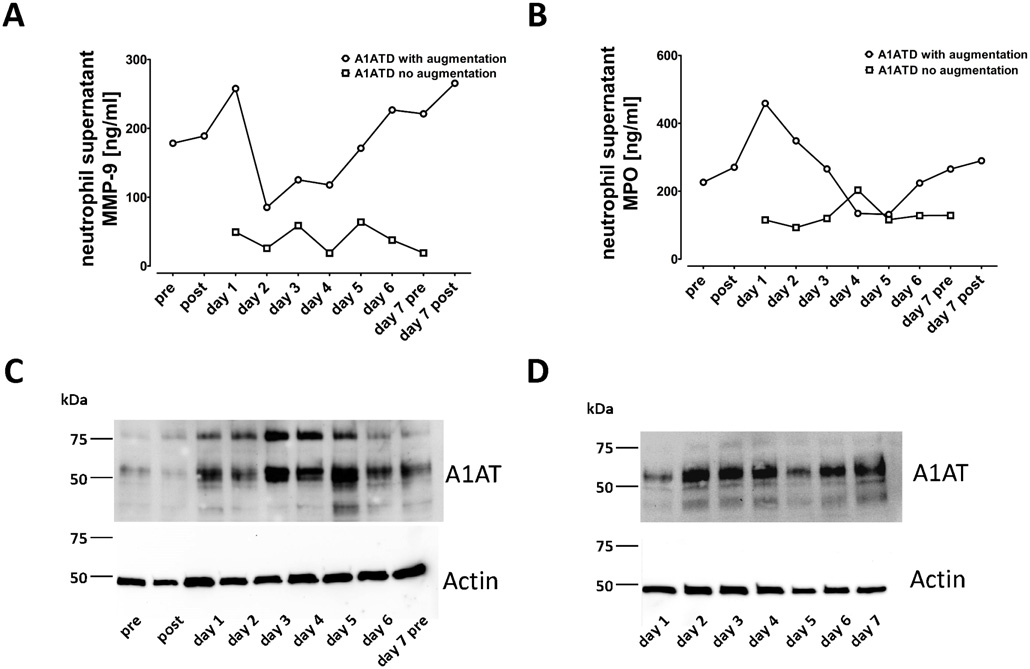
Analysis of MMP-9 and MPO in supernatants of neutrophil isolated from a single PiZZ patient. Neutrophils were isolated every day from one augmented PiZZ A1AT deficient-patient (GOLD stage III), and one non-augmented PiZZ A1AT deficient-patient (GOLD stage II). (***A***) matrix metalloproteinase 9 (MMP-9) and (***B***) myeloperoxidase (MPO) were analysed in the supernatants of 1x10^6^ cells/ ml (4 hours, 5% CO_2_, 37°C). Neutrophil-associated A1AT was analysed in augmented (***C***) and non-augmented (***D***) PiZZ A1AT deficient-patient by Western blot using polyclonal antibody against A1AT.

Blood neutrophils from one PiZZ patient (GOLD III, Table 1) treated with Prolastin were obtained every day during one week whereas twice on days one and seven: before (pre) and two hours after (post) A1AT therapy. Neutrophils were incubated at 37°C, 5% CO_2_ for 4 h and concentrations of released MMP-9 and MPO were determined in cell culture supernatants. As shown in Fig. 3A, the highest level of MMP-9 was measured one day after therapy, then decreased at a minimum at days three and four, and started to rise again at days five, six and seven to the levels measured before starting the therapy. Similarly, the highest level of MPO release was measured at day one post A1AT therapy and returned to basal levels by day three. In contrast to MMP-9, levels of MPO remained low until day seven (Fig. 3B). In supernatants of neutrophils isolated from non-augmented PiZZ patient (GOLD II, Table 1) MMP-9 and MPO release did not change during one week (Fig. 3A and B). Immunoelectrophoretic patterns of neutrophil-associated A1AT examined by Western blots revealed native and complexed forms of A1AT in the patient treated with Prolastin (Fig. 3C) whereas the non-treated patient showed one immunoreactive band typical for native A1AT (Fig. 3D). Notably, in the augmented patient strongest immunoreactivity for neutrophil-associated A1AT was observed at days three, four and five (Fig. 3C).

### Effects of Prolastin on MMP-9 and MPO release from isolated neutrophils, *in vitro*

**Fig 4:**
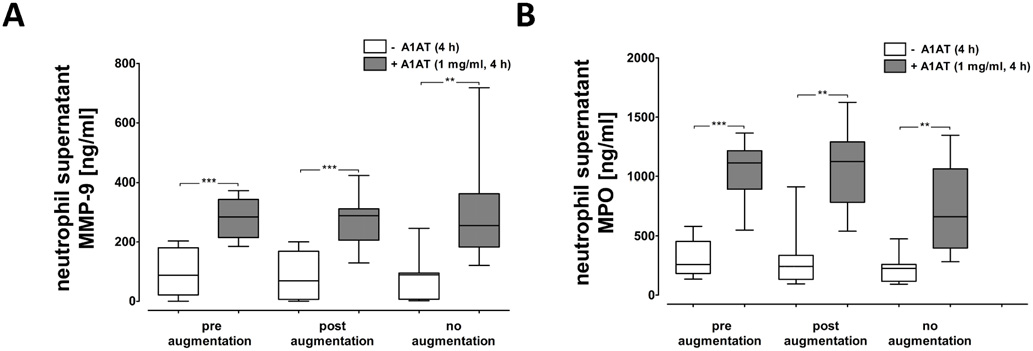
Analysis of MMP-9 and MPO in supernatants of neutrophils treated with Prolastin. Neutrophils were isolated from PiZZ A1AT deficient-patients before (pre, n = 8) and two hours after (post, n = 8) Prolastin therapy and from 8 PiZZ A1AT deficient-patients without Prolastin therapy. Quantification of the matrix metalloproteinase 9 (MMP-9) (**A**) myeloperoxidase (MPO) levels (**B**) was performed with or without pre-treatment of neutrophils (1x10^6^cells/ml) with Prolastin (1 mg/ml, 4 hours, 5% CO_2_, 37°C). Statistical significance between the groups is indicated as: *** p ≤ 0.001 and ** p ≤ 0.01.

In the next set of experiments, blood neutrophils were isolated from eight PiZZ patients before (pre) and two hours after (post) Prolastin therapy, and from eight PiZZ patients without therapy (Table 2). Neutrophils (1x10^7^cells/ml) were incubated alone or in the presence of Prolastin (1 mg/ml) at 37°C, 5% CO_2_ for 4 hours. As illustrated in Fig. 4A and B (open box plots), no significant difference was found between neutrophils isolated from patients before or two hours after A1AT therapy, or without augmentation therapy regarding the levels of MMP-9 and MPO. However, independent of whether neutrophils were obtained from treated or non-treated patients, exogenously added Prolastin significantly enhanced the release of MMP-9 and MPO (Fig. 4).

### Transient increase in MMP-9 release is associated with the presence of IgA in the Prolastin preparation

**Fig 5:**
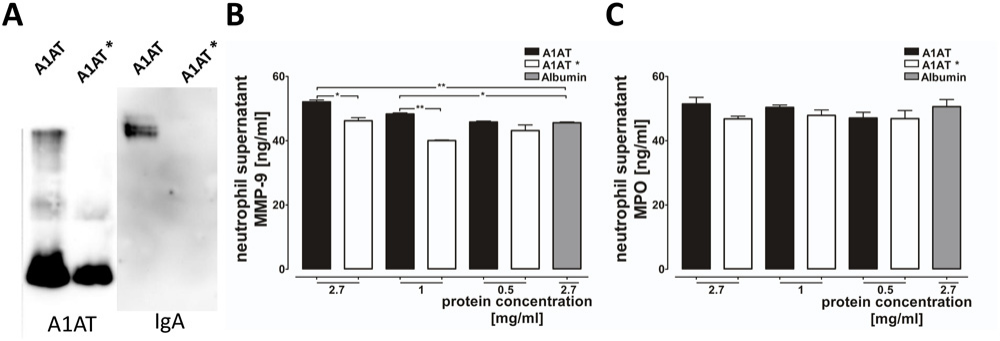
Analysis of MMP-9 and MPO in supernatants of neutrophils treated with Prolastin and Prolasin preparation without IgA. (*A*) Prolastin (A1AT) and Prolastin preparation without IgA (A1AT*) were separated by non-reducing polyacrylamid gel gelelectrophoresis followed by Western blot analysis with anti-A1AT and Anti-IgA antibodies. (*B*) Neutrophils were isolated from healthy controls (Pi MM, n = 3) and treated with different concentrations of A1AT, or A1AT* or human serum albumin (protein control) for two hours, at 37°C, 5% CO_2_. The concentration of 2.7 mg/ml mimics the average serum concentration post infusion of the augmented A1AT deficient patients. Quantification of the matrix metalloproteinase 9 (MMP-9, *B*) and myeloperoxidase (MPO, *C*) levels from cell culture supernatants were performed. Statistical significance between the groups is indicated as: ** p ≤ 0.009 and * p ≤ 0.02.

It is well known that Prolastin preparations contain minor amounts of IgA [23,24], which may trigger Fc receptor signaling and induce temporal neutrophil activation [25,26]. Therefore, we have re-purified Prolastin by using ultra-filtration and removed any traces of IgA (Fig. 5A). To further examine the effects of Prolastin on MMP-9 and MPO release freshly isolated neutrophils from healthy donors were treated for 2 hours with various concentrations of Prolastin or ultra-filtrated Prolastin (preparation completely lacking IgA) or 2.7 mg/ml human serum albumin (control). In comparison with ultra-filtrated Prolastin or albumin (control), Prolastin induced a significant MMP-9 release in a concentration dependent manner (Fig. 5 B). Remarkably under the same experimental conditions, Prolastin did not induce MPO release (Fig. 5C).

## Discussion

In this study we unexpectedly found that therapy with Prolastin causes a time-dependent modulation of serum MMP-9 and MPO levels. The therapy up-regulated serum levels of MMP-9 and MPO in an acute manner (within 2 hours) but after three days MMP-9 and MPO decreased below the levels measured before starting the therapy. In general, patients treated with Prolastin had significantly lower serum levels of MMP-9 and MPO than non-treated patients.

The close association between MMP-9 levels and high neutrophil numbers in COPD patients identify this cell type as a major source of MMP-9 [27,28]. Unlike *de novo* MMP-9 production by other cell types, such as macrophages, activated neutrophils release MMP-9 rapidly and independent of TIMP-1, as neutrophils do not produce TIMP-1 [29]. MMP-9 is formed in the later stages of neutrophil maturation and this proteinase contributes to neutrophil extravasation and stem cell mobilization via the degradation of basement membrane collagens [30,31]. Furthermore, observed correlation between increased MPO (marker of neutrophils degranulation) and increased MMP-9 concentration in serum of PiZZ patients (independently on augmentation therapy) implies that neutrophils are the main source of these inflammatory biomarkers. Therefore, we hypothesize that Prolastin-induced fluctuations in serum levels of MMP-9 and MPO result from its effect on neutrophil activation. Similar to the findings observed in serum, neutrophils isolated directly post therapy released more MMP-9 and MPO when neutrophils isolated three days post therapy. According to the Western blots, at this latter time point neutrophils had a high content of A1AT and A1AT occurred in several molecular forms. Some of these were higher molecular size than native A1AT, most likely representing complex with neutrophil proteases like elastase. Elastase is known to activate MMP-9 [32] therefore A1AT by inactivating elastase may be a physiological inhibitor of MMP-9 *in vivo*. This might also explain the lower serum MMP-9 levels observed in patients treated with Prolastin than those without this therapy.

To investigate the effects of Prolastin in more detail, we next treated freshly isolated human blood neutrophils with Prolastin, *ex vivo*. Once again, we found that 1 mg/ml Prolastin added to neutrophil cultures, within the first few hours promotes release of MMP-9 and MPO. Thus, our *ex vivo* data confirmed that therapy with Prolastin induces time-dependent changes in MMP-9 and MPO release from neutrophils.

With more than 25 years of experience Prolastin therapy is considered to be well tolerated and safe. However, this therapy is contraindicated in immunoglobulin A (IgA) deficient patients because preparations of A1AT may contain small amounts of IgA, which can induce IgA-related anaphylactic reactions [24,33]. It is also known that interaction of IgA with the myeloid IgA Fc receptor FcαRI (CD89) can induce various effector functions of leukocytes [34]. We speculated that there might be an association between the IgA impurity in Prolastin preparations and a short-term activation of neutrophils *in vivo* and *ex vivo*. Indeed, we found that Prolastin preparation contains small amount of IgA and used ultrafiltration to remove this impurity. As predicted, when neutrophils were treated with ultra-pure Prolastin for a short-term (2 hours) no induction in MMP-9 and MPO release was observed. Our results support the notion that IgA contamination might be responsible for a short-term neutrophil activation during therapy with Prolastin. This may be of significance in host response to therapy and may deserve further investigations.

Because neutrophils are the sole cells in the body that produce and secrete MMP-9 without its endogenous inhibitor TIMP-1, several studies point to a critical role for neutrophils and MMP-9 in blood vessel growth [34,35]. It is well known that MMP-9 degrades components of the extracellular matrix, facilitating tissue remodeling and thereby activating and liberating growth factors, such as vascular endothelial growth factor-A (VEGF-A) [36]. MMP-9 is also involved in the regulation of leukocytosis [28]. In support to the latter, we previously found that Prolastin induces a transient elevation of serum VEGF levels, now we additionally observed that it increases leukocyte numbers [20 PiZZ patients, mean (SD): age = 54.6±7.9 years and FEV_1_ = 40.8±14.6%: pre infusion 6.8±4.9 G leukocytes/L versus post infusion 7.11±1.9 G leukocytes/L, p = 0.0026]. Reduction in the level of VEGF has been implicated in the pathogenesis of pulmonary emphysema [37,38]. Airway administration of VEGF has been shown to improve lung maturation in mouse fetus without inducing angiogenesis [39]. Since the lung is exposed constantly to external stresses, induction of VEGF may help in the maintenance of lung repair and homeostasis.

A recent study by Bradley and coworkers addressed the contribution of MMP-9 during influenza virus pathogenesis and demonstrated that MMP-9- mediated neutrophil migration into the infected respiratory tract is required for viral clearance [40]. The necessity for MMP- 9-mediated immune cell migration and its role in immunopathology provides support for putative therapeutic benefits of Prolastin during virus infection. Indeed, results from several clinical cohorts show that A1AT therapy reduces number of exacerbations and slows-down the decline of lung function [41–44].

As a matter of fact, MMP-9 levels are elevated in patients with emphysema [20,45], cancer [46,47], diabetes [48], acute myocardial infarction [49,50] and other chronic conditions. Persistent elevation of MMP-9 is linked to disease progression [51]. As already mentioned above, following fast induction of MMP-9 and MPO release, Prolastin therapy in general lowers serum levels of MMP-9 and MPO relative to non-treated PiZZ patients. This finding is in accordance with a previous study demonstrating a reduction of MMP-9 on weekly treatment with augmentation therapy [52]. In addition to the effects on MMP-9, we previously found that Prolastin therapy significantly reduces serum levels of IL-8 [16] as well as the levels of TNF, IL-1, IL-6, MCP-1 in experimental models *in vitro* and *in vivo* [16,53,54].

Taken together, our findings support pleiotropic effects of Prolastin on inflammatory biomarkers of COPD. We appreciate that daily monitoring with solely one patient per group limits the significance but confirms the fluctuations found on day three in the treated patients and reflects the stability of these biomarkers in the non-treated patient group. The effect of long-term repetitive applications of Prolastin needs to be studied in a large multicenter cohort.

## References

[1] Voulgari F, Cummins P, Gardecki TI, Beeching NJ, Stone PC, et al (1982) Serum levels of acute phase and cardiac proteins after myocardial infarction, surgery, and infection. Br Heart J 48: 352–356. 6181800 10.1136/hrt.48.4.352 PMC481259

[2] Janciauskiene S (2001) Conformational properties of serine proteinase inhibitors (serpins) confer multiple pathophysiological roles. Biochim Biophys Acta 1535: 221–235. 11278163 10.1016/s0925-4439(01)00025-4

[3] Boskovic G, Twining SS (1997) Retinol and retinaldehyde specifically increase alpha1-proteinase inhibitor in the human cornea. Biochem J 322 (Pt 3): 751–756. 9148745 10.1042/bj3220751 PMC1218251

[4] Blanco I, de Serres FJ, Fernandez-Bustillo E, Lara B, Miravitlles M (2006) Estimated numbers and prevalence of PI*S and PI*Z alleles of alpha1-antitrypsin deficiency in European countries. Eur Respir J 27: 77–84. 16387939 10.1183/09031936.06.00062305

[5] Seersholm N, Kok-Jensen A (1998) Clinical features and prognosis of life time non-smokers with severe alpha 1-antitrypsin deficiency. Thorax 53: 265–268. 9741368 10.1136/thx.53.4.265 PMC1745189

[6] Kalsheker NA (2009) alpha1-Antitrypsin deficiency: best clinical practice. J Clin Pathol 62: 865–869. 10.1136/jcp.2008.063495 19783716

[7] Mahadeva R, Atkinson C, Li Z, Stewart S, Janciauskiene S, et al (2005) Polymers of Z alpha1-antitrypsin co-localize with neutrophils in emphysematous alveoli and are chemotactic in vivo. Am J Pathol 166: 377–386. 15681822 10.1016/s0002-9440(10)62261-4 PMC3278851

[8] Parmar JS, Mahadeva R, Reed BJ, Farahi N, Cadwallader KA, et al (2002) Polymers of alpha(1)-antitrypsin are chemotactic for human neutrophils: a new paradigm for the pathogenesis of emphysema. Am J Respir Cell Mol Biol 26: 723–730. 12034572 10.1165/ajrcmb.26.6.4739

[9] Alam S, Li Z, Janciauskiene S, Mahadeva R (2011) Oxidation of Z alpha1-antitrypsin by cigarette smoke induces polymerization: a novel mechanism of early-onset emphysema. Am J Respir Cell Mol Biol 45: 261–269. 10.1165/rcmb.2010-0328OC 20971880

[10] Mohanka M, Khemasuwan D, Stoller JK (2012) A review of augmentation therapy for alpha-1 antitrypsin deficiency. Expert Opin Biol Ther 12: 685–700. 10.1517/14712598.2012.676638 22500781

[11] Stockley RA, Miravitlles M, Vogelmeier C (2013) Augmentation therapy for alpha-1 antitrypsin deficiency: towards a personalised approach. Orphanet J Rare Dis 8: 149 10.1186/1750-1172-8-149 24063809 PMC3852071

[12] Parr DG, Dirksen A, Piitulainen E, Deng C, Wencker M, et al (2009) Exploring the optimum approach to the use of CT densitometry in a randomised placebo-controlled study of augmentation therapy in alpha 1-antitrypsin deficiency. Respir Res 10: 75 10.1186/1465-9921-10-75 19678952 PMC2740846

[13] Stockley RA, Parr DG, Piitulainen E, Stolk J, Stoel BC, et al (2010) Therapeutic efficacy of alpha-1 antitrypsin augmentation therapy on the loss of lung tissue: an integrated analysis of 2 randomised clinical trials using computed tomography densitometry. Respir Res 11: 136 10.1186/1465-9921-11-136 20920370 PMC2964614

[14] Stockley RA, Bayley DL, Unsal I, Dowson LJ (2002) The effect of augmentation therapy on bronchial inflammation in alpha1-antitrypsin deficiency. Am J Respir Crit Care Med 165: 1494–1498. 12045122 10.1164/rccm.2109013

[15] Nita I, Hollander C, Westin U, Janciauskiene SM (2005) Prolastin, a pharmaceutical preparation of purified human alpha1-antitrypsin, blocks endotoxin-mediated cytokine release. Respir Res 6: 12 15683545 10.1186/1465-9921-6-12 PMC549028

[16] Schmid ST, Koepke J, Dresel M, Hattesohl A, Frenzel E, et al (2012) The effects of weekly augmentation therapy in patients with PiZZ alpha1-antitrypsin deficiency. Int J Chron Obstruct Pulmon Dis 7: 687–696. 10.2147/COPD.S34560 23055718 PMC3468059

[17] Pinto-Plata V, Toso J, Lee K, Park D, Bilello J, et al (2007) Profiling serum biomarkers in patients with COPD: associations with clinical parameters. Thorax 62: 595–601. 17356059 10.1136/thx.2006.064428 PMC2117244

[18] Higashimoto Y, Iwata T, Okada M, Satoh H, Fukuda K, et al (2009) Serum biomarkers as predictors of lung function decline in chronic obstructive pulmonary disease. Respir Med 103: 1231–1238. 10.1016/j.rmed.2009.01.021 19249197

[19] Brajer B, Batura-Gabryel H, Nowicka A, Kuznar-Kaminska B, Szczepanik A (2008) Concentration of matrix metalloproteinase-9 in serum of patients with chronic obstructive pulmonary disease and a degree of airway obstruction and disease progression. J Physiol Pharmacol 59 Suppl 6: 145–152. 19218638

[20] Omachi TA, Eisner MD, Rames A, Markovtsova L, Blanc PD (2011) Matrix metalloproteinase-9 predicts pulmonary status declines in alpha1-antitrypsin deficiency. Respir Res 12: 35 10.1186/1465-9921-12-35 21429222 PMC3073899

[21] Stone H, McNab G, Wood AM, Stockley RA, Sapey E (2012) Variability of sputum inflammatory mediators in COPD and alpha1-antitrypsin deficiency. Eur Respir J 40: 561–569. 10.1183/09031936.00162811 22700846

[22] Al-Omari M, Korenbaum E, Ballmaier M, Lehmann U, Jonigk D, et al (2011) The acute phase protein, alpha1-antitrypsin, inhibits neutrophil calpain I and induces random migration. Mol Med. 10.2119/molmed.2011.00303 21494752 PMC3188872

[23] Kolarich D, Turecek PL, Weber A, Mitterer A, Graninger M, et al (2006) Biochemical, molecular characterization, and glycoproteomic analyses of alpha(1)-proteinase inhibitor products used for replacement therapy. Transfusion. 46(11):1959–77. 17076852 10.1111/j.1537-2995.2006.01004.x

[24] Cowden D, Fisher GE, Weeks RL (2005) A pilot study comparing the purity, functionality and isoform composition of alpha-1-proteinase inhibitor (human) products.Curr Med Res Opin. 21(6):877–83. 15969888 10.1185/030079905X46395

[25] Wehrli M, Cortinas-Elizondo F, Hlushchuk R, Daudel F, Villiger PM, et al (2014) Human IgA Fc Receptor FcαRI (CD89) Triggers Different Forms of Neutrophil Death Depending on the Inflammatory Microenvironment. J Immunol 193(11):5649–59 10.4049/jimmunol.1400028 25339672

[26] Otten MA1, van Egmond M (2004) The Fc receptor for IgA (FcalphaRI, CD89). Immunol Lett. 92(1–2):23–31. 15081523 10.1016/j.imlet.2003.11.018

[27] Segura-Valdez L, Pardo A, Gaxiola M, Uhal BD, Becerril C, et al (2000) Upregulation of gelatinases A and B, collagenases 1 and 2, and increased parenchymal cell death in COPD. Chest 117: 684–694. 10712992 10.1378/chest.117.3.684

[28] Vernooy JH, Lindeman JH, Jacobs JA, Hanemaaijer R, Wouters EF (2004) Increased activity of matrix metalloproteinase-8 and matrix metalloproteinase-9 in induced sputum from patients with COPD. Chest 126: 1802–1810. 15596677 10.1378/chest.126.6.1802

[29] Masure S, Proost P, Van Damme J, Opdenakker G (1991) Purification and identification of 91-kDa neutrophil gelatinase. Release by the activating peptide interleukin-8. Eur J Biochem 198: 391–398. 1645657 10.1111/j.1432-1033.1991.tb16027.x

[30] Opdenakker G, Fibbe WE, Van Damme J (1998) The molecular basis of leukocytosis. Immunol Today 19: 182–189. 9577095 10.1016/s0167-5699(97)01243-7

[31] Pruijt JF, Fibbe WE, Laterveer L, Pieters RA, Lindley IJ, et al (1999) Prevention of interleukin-8-induced mobilization of hematopoietic progenitor cells in rhesus monkeys by inhibitory antibodies against the metalloproteinase gelatinase B (MMP-9). Proc Natl Acad Sci U S A 96: 10863–10868. 10485917 10.1073/pnas.96.19.10863 PMC17974

[32] Ferry G, Lonchampt M, Pennel L, de Nanteuil G, Canet E, et al (1997) Activation of MMP-9 by neutrophil elastase in an in vivo model of acute lung injury. FEBS Lett. 402(2–3):111–5. 9037177 10.1016/s0014-5793(96)01508-6

[33] Dickens JA, Lomas DA (2011) Why has it been so difficult to prove the efficacy of alpha-1-antitrypsin replacement therapy? Insights from the study of disease pathogenesis. Drug Des Devel Ther. 5:391–405 10.2147/DDDT.S14018 21966212 PMC3180514

[34] Opdenakker G, Van den Steen PE, Dubois B, Nelissen I, Van Coillie E, et al (2001) Gelatinase B functions as regulator and effector in leukocyte biology. J Leukoc Biol 69: 851–859. 11404367

[35] Ardi VC, Kupriyanova TA, Deryugina EI, Quigley JP (2007) Human neutrophils uniquely release TIMP-free MMP-9 to provide a potent catalytic stimulator of angiogenesis. Proc Natl Acad Sci U S A 104: 20262–20267. 18077379 10.1073/pnas.0706438104 PMC2154419

[36] Van den Steen PE, Dubois B, Nelissen I, Rudd PM, Dwek RA, et al (2002) Biochemistry and molecular biology of gelatinase B or matrix metalloproteinase-9 (MMP-9). Crit Rev Biochem Mol Biol 37: 375–536. 12540195 10.1080/10409230290771546

[37] Kasahara Y, Tuder RM, Taraseviciene-Stewart L, Le Cras TD, Abman S, et al (2000) Inhibition of VEGF receptors causes lung cell apoptosis and emphysema. J Clin Invest 106: 1311–1319. 11104784 10.1172/JCI10259 PMC387249

[38] Tuder RM, Zhen L, Cho CY, Taraseviciene-Stewart L, Kasahara Y, et al (2003) Oxidative stress and apoptosis interact and cause emphysema due to vascular endothelial growth factor receptor blockade. Am J Respir Cell Mol Biol 29: 88–97. 12600822 10.1165/rcmb.2002-0228OC

[39] Compernolle V, Brusselmans K, Acker T, Hoet P, Tjwa M, et al (2002) Loss of HIF-2alpha and inhibition of VEGF impair fetal lung maturation, whereas treatment with VEGF prevents fatal respiratory distress in premature mice. Nat Med 8: 702–710. 12053176 10.1038/nm721

[40] Bradley LM, Douglass MF, Chatterjee D, Akira S, Baaten BJ (2012) Matrix metalloprotease 9 mediates neutrophil migration into the airways in response to influenza virus-induced toll-like receptor signaling. PLoS Pathog 8: e1002641 10.1371/journal.ppat.1002641 22496659 PMC3320598

[41] Seersholm N, Wencker M, Banik N, Viskum K, Dirksen A, et al (1997) Does alpha1-antitrypsin augmentation therapy slow the annual decline in FEV1 in patients with severe hereditary alpha1-antitrypsin deficiency? Wissenschaftliche Arbeitsgemeinschaft zur Therapie von Lungenerkrankungen (WATL) alpha1-AT study group. Eur Respir J 10: 2260–2263. 9387950 10.1183/09031936.97.10102260

[42] (1998) Survival and FEV1 decline in individuals with severe deficiency of alpha1-antitrypsin. The Alpha-1-Antitrypsin Deficiency Registry Study Group. Am J Respir Crit Care Med 158: 49–59. 9655706 10.1164/ajrccm.158.1.9712017

[43] Lieberman J (2000) Augmentation therapy reduces frequency of lung infections in antitrypsin deficiency: a new hypothesis with supporting data. Chest 118: 1480–1485. 11083705 10.1378/chest.118.5.1480

[44] Wencker M, Fuhrmann B, Banik N, Konietzko N (2001) Longitudinal follow-up of patients with alpha(1)-protease inhibitor deficiency before and during therapy with IV alpha(1)-protease inhibitor. Chest 119: 737–744. 11243951 10.1378/chest.119.3.737

[45] Boschetto P, Quintavalle S, Zeni E, Leprotti S, Potena A, et al (2006) Association between markers of emphysema and more severe chronic obstructive pulmonary disease. Thorax 61: 1037–1042. 16769715 10.1136/thx.2006.058321 PMC2117071

[46] Roy R, Yang J, Moses MA (2009) Matrix metalloproteinases as novel biomarkers and potential therapeutic targets in human cancer. J Clin Oncol 27: 5287–5297. 10.1200/JCO.2009.23.5556 19738110 PMC2773480

[47] Kessenbrock K, Plaks V, Werb Z (2010) Matrix metalloproteinases: regulators of the tumor microenvironment. Cell 141: 52–67. 10.1016/j.cell.2010.03.015 20371345 PMC2862057

[48] Kowluru RA, Zhong Q, Santos JM (2012) Matrix metalloproteinases in diabetic retinopathy: potential role of MMP-9. Expert Opin Investig Drugs 21: 797–805. 10.1517/13543784.2012.681043 22519597 PMC3802521

[49] Wang KF, Huang PH, Chiang CH, Hsu CY, Leu HB, et al (2013) Usefulness of plasma matrix metalloproteinase-9 level in predicting future coronary revascularization in patients after acute myocardial infarction. Coron Artery Dis 24: 23–28. 10.1097/MCA.0b013e32835aab4a 23151854

[50] Shand JA, Menown IB, McEneaney DJ (2010) A timely diagnosis of myocardial infarction. Biomark Med 4: 385–393. 10.2217/bmm.10.16 20550472

[51] Aldonyte R, Eriksson S, Piitulainen E, Wallmark A, Janciauskiene S (2004) Analysis of systemic biomarkers in COPD patients. Copd 1: 155–164. 17136983 10.1081/copd-120030828

[52] Bergin DA, Reeves EP, Hurley K, Wolfe R, Jameel R, et al (2014) The circulating proteinase inhibitor alpha-1 antitrypsin regulates neutrophil degranulation and autoimmunity. Sci Transl Med 6: 217ra211. 10.1126/scitranslmed.3007116 24382893

[53] Jonigk D, Al-Omari M, Maegel L, Muller M, Izykowski N, et al (2013) Anti-inflammatory and immunomodulatory properties of alpha1-antitrypsin without inhibition of elastase. Proc Natl Acad Sci U S A 110: 15007–15012. 10.1073/pnas.1309648110 23975926 PMC3773761

[54] Ozeri E, Mizrahi M, Shahaf G, Lewis EC (2012) alpha-1 antitrypsin promotes semimature, IL-10-producing and readily migrating tolerogenic dendritic cells. J Immunol 189: 146–153. 10.4049/jimmunol.1101340 22634621

